# *Anaplasma marginale* Infection of *Dermacentor andersoni* Primary Midgut Cell Culture Is Dependent on Fucosylated Glycans

**DOI:** 10.3389/fcimb.2022.877525

**Published:** 2022-05-31

**Authors:** Rubikah Vimonish, Janaina Capelli-Peixoto, Wendell C. Johnson, Hala E. Hussein, Naomi S. Taus, Kelly A. Brayton, Ulrike G. Munderloh, Susan M. Noh, Massaro W. Ueti

**Affiliations:** ^1^ Program in Vector-borne Diseases, Department of Veterinary Microbiology and Pathology, Washington State University, Pullman, WA, United States; ^2^ Animal Diseases Research Unit, United States Department of Agriculture-Agricultural Research Service (USDA-ARS), Pullman, WA, United States; ^3^ Department of Entomology, Faculty of Science, Cairo University, Giza, Egypt; ^4^ School of Public Health, Division of Environmental Health Sciences, University of Minnesota, Minneapolis, MN, United States; ^5^ The Paul G. Allen School for Global Animal Health, Washington State University, Pullman, WA, United States

**Keywords:** *Dermacentor andersoni*, midgut cells, primary cell culture, fucosyltransferase, glycans, *Anaplasma marginale*, tick

## Abstract

Tick midgut is the primary infection site required by tick-borne pathogens to initiate their development for transmission. Despite the biological significance of this organ, cell cultures derived exclusively from tick midgut tissues are unavailable and protocols for generating primary midgut cell cultures have not been described. To study the mechanism of *Anaplasma marginale*-tick cell interactions, we successfully developed an *in vitro Dermacentor andersoni* primary midgut cell culture system. Midgut cells were maintained for up to 120 days. We demonstrated the infection of *in vitro* midgut cells by using an *A. marginale omp10::himar1* mutant with continued replication for up to 10 days post-infection. *Anaplasma marginale* infection of midgut cells regulated the differential expression of tick α-(1,3)-fucosyltransferases A1 and A2. Silencing of α-(1,3)-fucosyltransferase A2 in uninfected midgut cells reduced the display of fucosylated glycans and significantly lowered the susceptibility of midgut cells to *A. marginale* infection, suggesting that the pathogen utilized core α-(1,3)-fucose of N-glycans to infect tick midgut cells. This is the first report using *in vitro* primary *D. andersoni* midgut cells to study *A. marginale*-tick cell interactions at the molecular level. The primary midgut cell culture system will further facilitate the investigation of tick-pathogen interactions, leading to the development of novel intervention strategies for tick-borne diseases.

## Introduction

Ticks are ectoparasites and pathogen vectors that can transmit a variety of bacteria, viruses, and protozoan parasites to both humans and animals (Brites-Neto et al., 2015). However, transmission prevention measures are limited due to the lack of understanding of the tick-pathogen interface (Rego et al., 2019). The tick midgut epithelium is the target site for the initiation of tick-borne pathogen transmission (Sonenshine and Macaluso, 2017). The epithelium of the midgut diverticula of fasting ticks is composed of a monolayer of resting digestive cells, degenerative digestive cells, and stem cells (Starck et al., 2018). *In vivo* studies of the physiology of the midgut epithelium and the mechanisms of pathogen infection are challenging. To understand the mechanisms of tick-pathogen interaction, studies of the infection at the cellular and molecular levels are needed.

The luminal face of the insect midgut epithelium is coated with a dense array of glycoconjugates that act as a “glycan receptor buffet” for pathogen interactions (Dinglasan and Jacobs-Lorena, 2005). Pathogen carbohydrate-binding proteins utilize arthropod host midgut glycans as attachment receptors for the invasion of midgut epithelial cells (Dinglasan and Jacobs-Lorena, 2005). Unfortunately, little is known regarding the glycobiology of tick cells. Currently, the study of tick glycobiology has been limited to the development of α-gal-specific IgE and hypersensitivity reactions in humans (Cabezas-Cruz et al., 2018; Crispell et al., 2019; Sharma et al., 2021) and the interaction between *Anaplasma phagocytophilum* and *Ixodes* tick cell glycans (Pedra et al., 2010; Seidman et al., 2015).

Invertebrate glycans are made by linking monosaccharides such as glucose, mannose, galactose, N-acetylglucosamine (GlcNAc), N-acetylgalactosamine (GalNAc), xylose, fucose, N-acetylneuraminic acid (NeuAc), N-glycoylneuraminic acid (NeuGc), glucuronic acid (GlcA), and iduronic acid. Glycans are attached to proteins through an enzymatic process called glycosylation (Zhu et al., 2019). Ninety percent of glycoproteins have N-linked glycosylation, where the glycan binds to the amino group of asparagine residues in the protein (Apweiler et al., 1999). Fucosylation is the process whereby fucose sugars are incorporated into N-glycans by fucosyltransferases (Becker and Lowe, 2003). A number of pathogens utilize fucosylated N-glycans during colonization of human epithelial cells, including *Helicobacter pylori* (Ilver et al., 1998), Norovirus (Chen et al., 2011), *Vibrio cholera* (Heim et al., 2019), and *Salmonella enterica* serovar Typhimurium (Suwandi et al., 2019). *Anaplasma phagocytophilum*, a bacterial pathogen of humans and domestic animals, requires α-(1,3)-fucosylation in its tick vector (*Ixodes* spp.) for colonization (Pedra et al., 2010). *Anaplasma* spp. have a conserved outer membrane protein that interacts with host cell α-(1,3)-fucosylated N-glycans as demonstrated by using both tick embryonic and mammalian cell cultures (Seidman et al., 2015; Hebert et al., 2017).

The present study is focused on determining if fucosylated glycan mediates interactions between *A. marginale* and its invertebrate host *D. andersoni*. *Anaplasma marginale* is the primary etiological agent of bovine anaplasmosis which is a significant tick-borne disease of livestock. This bacterium invades its vector tick midgut for initial development during acquisition, which is required for successful tick colonization and ongoing transmission (Kocan et al., 1992). To study *A. marginale* and tick midgut cell interactions at the cellular and molecular levels, an *in vitro* tick midgut cell culture is required. Most currently available tick cell lines were isolated from embryonated eggs containing multiple cell types which may not include differentiated midgut cells (Bell-Sakyi, 1991; Munderloh et al., 1994; Bell-Sakyi et al., 2018; Lima-Duarte et al., 2021; Salata et al., 2021). Heretofore, the lack of available tick cell culture systems derived from midgut has precluded the *in vitro* investigation of *A. marginale*-tick midgut cell interactions. In the present study, we first developed primary tick midgut cell cultures derived from male *D. andersoni*. Male ticks have been shown to be epidemiologically relevant for the transmission of *A. marginale* (Eriks et al., 1993; Futse et al., 2003; Ueti et al., 2009). Secondly, we demonstrated that primary tick midgut cells are permissive for *A. marginale* infection, and finally, we demonstrated that fucosylated N-glycans were required for *A. marginale* infection of *D. andersoni* midgut cell cultures. A better understanding of how *A. marginale* utilizes α-(1,3)-fucosylation may lead to the development of novel therapeutic interventions against bovine anaplasmosis.

## Materials and Methods

### Primary Tick Midgut Cell Culture

Specific pathogen-free ticks from the *D. andersoni* Reynolds Creek colony (Scoles et al., 2007) were used to develop primary midgut cell cultures. Nymphs were applied under a cloth patch on the back of uninfected calves and allowed to feed to repletion. Replete nymphs were incubated at 26°C and 94% relative humidity to molt to adults. Adult ticks were maintained in an incubator at 15°C and 94% relative humidity without feeding for approximately one year. This study was approved (protocol # 2020-60) by the Institutional Animal Care and Use Committee of the University of Idaho (Moscow, ID, USA).

Male ticks were surface sterilized by immersion in successive one-minute washes of 70% ethanol and 0.1% sodium hypochlorite as previously described for lepidopteran species (Garcia et al., 2001). Hemolymph was collected during tick dissection and the pH determined by placing a small volume on pH test strips (Thermo Fisher Scientific, Waltham, MA). The ticks were dissected in ice cold wash solution (Hank’s balanced salt solution without Ca^2+^ and Mg^2+^ (Gibco, Waltham, MA), 1X Antibiotic Antimycotic solution (Sigma-Aldrich, St. Louis, MO), and 50 μg/ml gentamicin (Sigma-Aldrich)). Tick midguts were removed and rinsed twice with wash solution. Tick midguts were then placed in a digestion buffer solution containing 800 CDU/ml collagenase type XI (Sigma-Aldrich), 1% v/v fetal bovine serum (Thermo Fisher Scientific, Waltham, MA), and 0.5 mM dithiothreitol (DTT) (Thermo Fisher Scientific) in Hank’s balanced salt solution without Ca^2+^ and Mg^2+^ (Gibco). Digestion took place in a 37°C incubator with shaking at 180 rpm for 90 min. Following digestion, the midgut cells were released from the tissue by gently pipetting with a wide-bore pipette, followed by filtering through a 70 µm cell strainer (Thermo Fisher Scientific). The filtrate was centrifuged at 200xg for 10 min and pelleted cells were washed twice with wash solution to remove collagenase. The number of viable cells was determined by the trypan blue exclusion test using a hemocytometer as previously described (Strober, 2001).

Viable midgut cells (3x10^5^ cells/ml) were suspended in 1 ml Hink’s TNM-FH insect medium (Sigma-Aldrich) containing 40 mg/ml AlbuMAX™ II (Gibco), 1 µM 20-hydroxyecdysone (20-HE) (Sigma-Aldrich), 100 units/ml penicillin, 100 µg/ml streptomycin and 250 ng/ml amphotericin B (Sigma-Aldrich), and 50 μg/ml gentamicin (Sigma-Aldrich) with a final pH of 7. The cell suspension was transferred to 24-well cell culture plates (Thermo Fisher Scientific) and placed in a humidified incubator at 34°C with 5% CO_2_. Culture medium containing antibiotic and antimycotic was replaced twice a week. Observations were made daily to examine midgut cell cultures over a period of 4 months with a Leica IX70 inverted microscope with LAS-X software (Leica Microsystems, Buffalo Grove, IL). To test attachment of cells to culture plates, bovine collagen I solution was diluted in sterile PBS at a concentration of 3 mg/ml and 100 μl added to each well. The plates were allowed to air-dry at room temperature. Dried coated plates were sterilized by rinsing with 70% ethanol before introducing the midgut cell suspension.

### Determining the Viability of Tick Midgut Cells

Tick midgut cells (1x10^3^ cells/ml) were suspended in PBS containing 0.1% BSA and an equal volume of 10μM 5(6)-cFDA in PBS/0.1% BSA (Bio-Rad, Hercules, CA) added to the cell suspension. Cell suspensions were gently mixed and incubated in the dark at 37°C for 15 min. The reactions were stopped by adding Hink’s TNM-FH insect medium and centrifuging at 200xg for 10 min. The cells were washed, and pellets suspended in Hink’s TNM-FH insect medium. The 5(6)-cFDA stained cells were stained with a cell-permeant nuclear stain, Hoechst 33342 (NucBlue™ Live ReadyProbes™ Reagent, Invitrogen, Waltham, MA, USA). Fluorescence images were obtained using a Leica IX70 inverted microscope with LAS-X software (Leica Microsystems).

### Determining Susceptibility of Primary Midgut Cells for *A. marginale* Infection

Primary cell cultures were established without antibiotics or antimycotics and maintained for 2 weeks. A tick-cell-free, Virginia strain-*A. marginale omp10::himar1* (Crosby et al., 2014) inoculum was prepared by passing heavily infected DAE100T cells derived from embryonic *D. andersoni* (Simser et al., 2001) through a 27-gauge needle to rupture the cells and release the bacteria. The *A. marginale omp10::himar1* was stored in Sucrose-Phosphate-Glutamate (SPG) buffer at -80°C. The SPG buffer, pH 7.2, consisted of 3.2 mM sodium phosphate monobasic, 7.2 mM sodium phosphate dibasic, 250 mM sucrose, 5mM L-glutamic acid (Sigma-Aldrich) in culture grade water and sterilized through a 0.22-micron filter. *Anaplasma marginale omp10::himar1* stock was thawed and filtered through a 5.0-micron pore size filter and centrifuged at 12,000xg for 7 min. A bacterial pellet of 3.4x10^5^ bacteria as determined by qPCR as previously described (Scoles et al., 2007) was suspended in 100 µl Hink’s TNM-FH insect medium and inoculated into individual culture wells. Culture medium was 1 ml of Hink’s TNM-FH insect medium containing 40 mg/ml of AlbuMAX™ II (Gibco) and 1 µM of 20-HE (Sigma-Aldrich) with a final pH of 7. Cultures were placed in a humidified incubator at 34°C with 5% CO_2_ for 10 days. Culture medium without antibiotic and antimycotic was replaced with fresh medium every 12-24 h. Infection of primary midgut cells was determined at 0 h, 24 h, 48 h, 72 h, 96 h, 120 h, and 10 days post-infection by fluorescent images obtained using a Leica IX70 inverted microscope with LAS-X software (Leica Microsystems).

### Confirmation of *A. marginale* Infection in Midgut Cell Culture by Immunofluorescence Assay

Midgut cells infected with *A. marginal omp10::himar1* were harvested and centrifuged at 200xg for 10 min. The cell pellet was washed and suspended in 400 µl of PBS and cells immobilized onto microscope slides (Rite-One™, Waltham, MA) using a Cytospin 4 cytocentrifuge (Thermo Fisher Scientific) at 1,000 rpm for 10 min. Slides were air-dried overnight at room temperature and fixed for 10 min in cool acetone. Fixed cells were blocked with 10% (v/v) goat serum (Gibco) for 30 min prior to incubation with monoclonal antibodies (10 µg/ml) raised against *A. marginale* major surface protein 2 (Msp2), AnaR49A1 (Ueti et al., 2009), or a *Trypanosoma brucei* protein, Tryp1a (Scoles et al., 2007; Ueti et al., 2009). Antibodies were diluted in PBS with 0.1% Tween 20 (PBST) and 1% BSA, added to slides, and incubated in a humidified chamber overnight at 4°C. After washing in PBST, goat anti-mouse Alexa Fluor 594 secondary antibody (Invitrogen) (5µg/ml) diluted in PBST/1% BSA was applied for one hour in the dark, at room temperature. The slides were washed three times in PBST. Cells were mounted using ProLong™ Gold Antifade Mountant containing DAPI (Invitrogen). Cells were examined using a Leica IX70 inverted microscope and fluorescence images were obtained using LAS-X software (Leica Microsystems).

### Identification of Fucosyltransferase Genes

Nucleotide sequences obtained from the Transcriptome Shotgun Assembly (TSA) database of *D. variabilis* (https://www.ncbi.nlm.nih.gov/nuccore/?term=dermacentor+variabilis+TSA) and the Sequence Read Archive (SRA) database of *D. andersoni* (https://www.ncbi.nlm.nih.gov/sra, accession no. SRX841407, SRX841406, SRX841365, SRX841363, SRX841359, SRX841353, SRX841346, SRX841324, SRX841262, SRX841240, SRX841234, SRX841229, SRX841222, SRX841215, SRX841167, SRX841166, SRX841134, SRX841115, SRX841106, SRX608566, SRX608565, SRX608563, SRX608541, SRX608542, SRX608552, SRX608554, SRX608555, SRX608558, SRX608559, SRX608561, SRX608533, SRX608301, SRX608300, SRX608299, SRX608298, SRX608297, SRX608296, SRX608295, SRX608294, SRX608292, SRX608291, SRX608290, SRX608289, SRX599931, SRX599930, SRX599929, SRX540760, SRX540759, SRX495490, SRX174800, SRX174799, SRX174798) in NCBI were assembled and aligned against the Expressed Sequence Tag (EST) database of *Rhipicephalus* spp (https://blast.ncbi.nlm.nih.gov/Blast.cgi?PROGRAM=blastn&PAGE_TYPE=BlastSearch&LINK_LOC=blasthome) fucosyltransferase gene sequences at NCBI. *Dermacentor andersoni* male ticks were dissected in Hank’s balanced salt solution (Gibco) and midguts collected in RNA Later solution (Thermo Fisher Scientific). Midgut epithelial cells from primary midgut cell cultures were collected in TRIzol™ Reagent (Invitrogen). Midguts or cultured cells were homogenized in 500 µl of TRIzol reagent with 100 µl of chloroform (Invitrogen) for phase separation. The aqueous phase was collected and 1 μl of glycogen (10 μg/ml) added to the samples. The RNA was precipitated with isopropyl alcohol, washed with 75% ethanol, and dissolved in DEPC-treated water. Extracted total RNA was treated with DNase I by using a DNase Kit (Thermo Fisher Scientific) following the manufacturer’s guideline. Total RNA (100 ng) was utilized for cDNA synthesis using a Superscript IІI™ cDNA Synthesis Kit (Thermo Fisher Scientific) following the manufacturer’s protocol. Oligonucleotide primer sequences were designed using PrimerQuest™ Tool (Integrated DNA Technologies) (**Table 1**) to amplify fucosyltransferase genes from cDNA derived from *D. andersoni* midguts. PCR reactions were performed in 20 μl containing 10 ng of synthesized cDNA, 10 μM of each primer set, 6 μl nuclease-free water and 10 μl RedTaq (Sigma-Aldrich). The amplification conditions consisted of denaturation at 95°C for 3 min, 35 repeated cycles at 95°C for 30 sec, 57°C-65°C (**Table 1**) for 30 sec and 72°C for 30 sec, with a final extension at 72°C for 7 min. Amplicons were resolved using 1% agarose gel electrophoresis. The PCR products were cloned into pCR 2.1-TOPO plasmids (Thermo Fisher Scientific) and transformed into TOP10 One Shot chemically competent *Escherichia coli* (Thermo Fisher Scientific) following the manufacturer’s guidelines. Transfected *E. coli* were plated on Luria Agar (Invitrogen) with 50 µg/ml of ampicillin and 10 positive clones per gene were grown in Luria Broth (Research Products International Corp. Mount Prospect, IL) with 50 µg/ml of ampicillin for 15 h at 37°C. Plasmid DNA was extracted using Miniprep Kit (Promega, Madison, WI) and sequenced (Eurofins, Lancaster, PA) using M13 forward and reverse primers. Nucleotide sequences were analyzed using DNASTAR™ Lasergene Genomics Suite Software (Thermo Fisher Scientific) (**Supplementary Figure 1**). Similarly, the *D. andersoni gapdh* nucleotide sequence was assembled by using the SRA database at NCBI and sequenced as described above (GenBank accession # OL791279). *Drosophila melanogaster* N-glycan linkage-specific fucosyltransferase sequences were used to identify putative orthologs in *D. andersoni* using the Basic Local Alignment Search Tool (BLAST) of NCBI.

**Table 1:** Primer sequences used in this study.

Oligonucleotide name	Tm (°C)	Forward (5′- 3′)	Reverse (5′- 3′)	Product size (bp)
DaFucT6	62	ATGGCTATGGGCTTGGGCAAGC	TCACTGCACGGGAAAGGGCAC	1638
DaFucTA1	65	ATGTTCCCGGCCAAGAAGCTCCGG	TCAGGTGAGCACGTACGCCACG	1404
DaFucTA2	60	ATGCGGGTGATCATGCTCCG	TTAGTAAGTAGGCACCCACACGGG	1190
DaFucTC1	57	ATGATTCTGAAGAGAAGGCGTCG	TCAATAAAGTTGTTTGGTTTCTGGATTCC	1206
DaFucTC2	60	ATGGCGGCCATCACCAAGAG	TTATTTCACCCACGTCCGACAGC	1134
DaFucTC3	65	ATGCGCGTGCCCTCGAGGT	TTAGTACCTAAACGTTTTCTTCCACG	1080
DaGapdh	60	ATGAGCGTGAAGATCGGCATCAACG	TTAGCCCCTGGACTTCATGTACTTGATC	1005
dsRNA-DaFucTA1	60	CCTCAGCTTCTGCACCGT	GTCGTAGCGCACGAACTTTT	700
dsRNA-DaFucTA2	60	CTCCGGCAGAAGAACTTCAA	TGCTTGGCTAGCTCCTTGAC	668
qPCR-DaFucTA1	62	GGCACGATTCGGACATAGTAG	GCGACCTTCTTGGTCTTGT	115
qPCR-DaFucTA2	62	GCCATCTTGGGTAGCAACT	CTTTAGTGACGTCGCAGTCC	148
qPCR-DaGapdh	62	GTGTCAACCACACTACCTACAA	GACATGAGTCCCTCGACAATG	127
qPCR-AMmsp5	55	CTTCCGAAGTTGTAAGTGAGGGCA	CTTATCGGCATGGTCGCCTAGTTT	202

### Determining the Expression of *D. andersoni* Fucosyltransferase TA1 and TA2 in Uninfected and *A. marginale* Infected Tick Midgut Cell Cultures

Uninfected primary cell cultures or cultures infected with *A. marginale omp10::himar1* were established and maintained for 2 weeks in medium without antibiotics or antimycotics. Total RNA was extracted from primary midgut cell cultures using TRIzol-chloroform as described above. The RNA samples were treated with DNase I (Thermo Fisher Scientific) following the manufacturer’s guideline. Total RNA (1,500 ng) of each sample was used to synthesize cDNA by using SuperScript™ III First-Strand Synthesis System (Invitrogen) following the manufacturer’s guideline. Gene specific primers for *DaFucTA1* and *DaFucTA2* were designed to amplify 115 bp and 148 bp fragments, respectively (**Table 1**). Midgut cell samples were normalized using qPCR targeting a 127 bp fragment of *D. andersoni gapdh* gene.

The qPCR reactions were performed in a CFX96™ Real-Time PCR Detection System (Bio-Rad) using SsoFast™ EvaGreen^®^ Supermix (Bio-Rad). Triplicate reactions were performed in 20 μl using 10 µM of each primer and 30 ng of cDNA as template. The cycling conditions consisted of an initial cycle at 95°C for 3 min, 40 cycles at 95°C for 30 sec, 60°C for 30 sec, and 72°C for 30 sec. Gene expression data were obtained by CFX Manager™ Software (Bio-Rad) and analyzed by the 2^-ΔΔCq^ method (Livak and Schmittgen, 2001).

### Synthesis of dsRNA

The nucleotide sequence of *DaFucTA2* was analyzed *in silico* to identify a double stranded RNA (dsRNA) template sequence with the highest number of performing siRNAs using E-RNAi software (German Cancer Research Center). The dsRNA template with opposing T7 promoters at the 5’ ends of each strand was generated by PCR with the T7 promoter appended to both PCR primers. The PCR product (668 bp) was purified using a High Pure PCR Purification Kit (Roche Molecular Biochemicals). The purified dsRNA template was used for transcription using a MEGAscript^®^ RNAi Kit (Ambion, Austin, TX) to produce dsRNA. To synthesize the non-tick specific control dsRNA, a 514 bp segment from the *Drosophila* nautilus gene (GenBank accession # M68897) was employed. One µg of dsRNA samples was analyzed by 1% agarose gel electrophoresis for the integrity and efficiency of duplex formation as well as the elimination of ssRNA and dsDNA following nuclease digestion. Purified dsRNA concentration was quantified by spectrophotometer nd-1000 (Thermo Fisher Scientific) and stored at -20°C until *in vitro* transfection.

### dsRNA Transfection of Primary Midgut Cell Cultures

Primary midgut cells were diluted to a final concentration of 3x10^5^ cells/ml in Hink’s TNM-FH insect medium with AlbuMAX™ II (Gibco) and plated in 24-well cell culture plates (Thermo Fisher Scientific) 24 h prior to use and incubated at 34°C with 5% CO_2_. One hundred and fifty μg of dsRNA was incubated in 100 µl of TNM-FH insect (Sigma-Aldrich) medium containing 10µl of FuGENE 6 Transfection Reagent (Promega) at room temperature for 15 min. For dsRNA derived from DaFucTA2, 150 μg of dsRNA corresponded to 2.2x10^14^ copies of dsRNAs as previously described (Bifano et al., 2014). Aliquots of 100 μl of transfection mix were added to the wells followed by agitation. The cells were incubated at 34°C for 48 h. Control cells received an equal concentration of dsRNA-*Drosophila* or transfection reagent and dsRNA elution buffer. Gene knockdown efficiency was determined at 48 h post-knockdown using qRT-PCR targeting *DaFucTA2* as described above. At 48 h post-knockdown, cells were exposed to 5.4x10^4^ *A. marginale omp10::himar1.* At 72 h post-knockdown, gene expression was measured by qRT-PCR targeting *DaFucTA1*, *DaFucTA2* or *A. marginale msp5* (**Table 1**) as described above. *Dermacentor andersoni gapdh* gene was used for normalization. Gene expression data were obtained by CFX Manager™ Software (Bio-Rad) and analyzed by the 2^-ΔΔCq^ method.

### Detection of Core α-(1,3)-Fucose

A midgut cell culture was suspended, and cells immobilized onto microscope slides (Rite-One™) by using a Cytospin 4 cytocentrifuge at 1,000 rpm for 10 min. Slides were dried overnight at room temperature and fixed for 10 min in cool acetone. Fixed cells were blocked with 10% (v/v) goat serum (Gibco) for 30 min prior to incubation with anti-HRP antibody (Sigma-Aldrich) at a dilution of 1:500 as described above. Goat anti-mouse IgG1 CF™488A (Sigma-Aldrich) (5µg/ml) was used as the secondary antibody. Cells were mounted using ProLong™ Gold Antifade Mountant containing DAPI (Invitrogen). Cells were examined using a Leica IX70 inverted microscope and fluorescence images were obtained using LAS-X software (Leica Microsystems).

### Statistical Analyses

Gene expression is displayed as means ± standard errors. Standard error for the 2^-ΔΔCq^ values were calculated using Microsoft Excel (2019). Unpaired t-test was performed using the GraphPad QuickCalcs software (GraphPad Software, La Jolla, CA) to compare the gene expression and *A. marginale* infection.

## Results

### Primary Midgut Cell Culture

**Figure 1:**
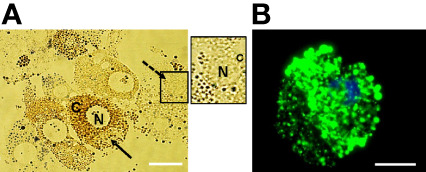
Isolated *Dermacentor andersoni* midgut cells in culture. **(A)** Midgut cells in culture immediately after dissociation; N, Nucleus; C, Cytoplasm, solid arrow indicates a digestive cell and dotted arrow indicate a non-digestive-like cell. Boxed area on the right depicts the non-digestive-like cell at a higher resolution. **(B)** Viable digestive cell. A cell stained with 5(6)-cFDA and Hoechst 33342. Green: viable cytoplasm, Blue: nucleus. Scale bar: 20µm.

A combination of collagenase, DTT, and fetal calf serum resulted in dissociation of *D. andersoni* midgut tissues into individual cells. In culture, the freshly dissociated cells contained two major cell types (**Figure 1A**) resembling digestive cells that were 18 µm to 30 µm in diameter and non-digestive like cells that were 6 µm to 10 µm in diameter. The larger cells (solid arrow) contained a dense dark, granular cytoplasm which appeared to consist of hemosomes and digestive vesicles. The smaller cells (dotted arrow) had a large nucleus with little cytoplasm. Following the dissociation procedure, viable midgut cells were visualized under fluorescence microscopy (**Figure 1B**).

To test the compatibility of the culture medium, the pH of *D. andersoni* unfed male tick hemolymph was determined and found to be between 6.5-7. Isolated cells were suspended in culture medium with a pH of 7 and distributed into culture plates. Isolated tick midgut cells loosely adhered to the cell culture plate. These cells varied in size with an agranular cytoplasm containing a large irregular nucleus. Aggregates of midgut cells were often observed as flat sheets. Treating cell culture plates with bovine collagen I promoted the attachment of midgut cells to the bottom of the wells. We noticed that the smaller non-digestive- and large digestive-like cells remained alive in *in vitro* cultures for up to four months.

### Determining the Susceptibility of Primary Midgut Cell Cultures to *A. marginale* Infection

**Figure 2:**
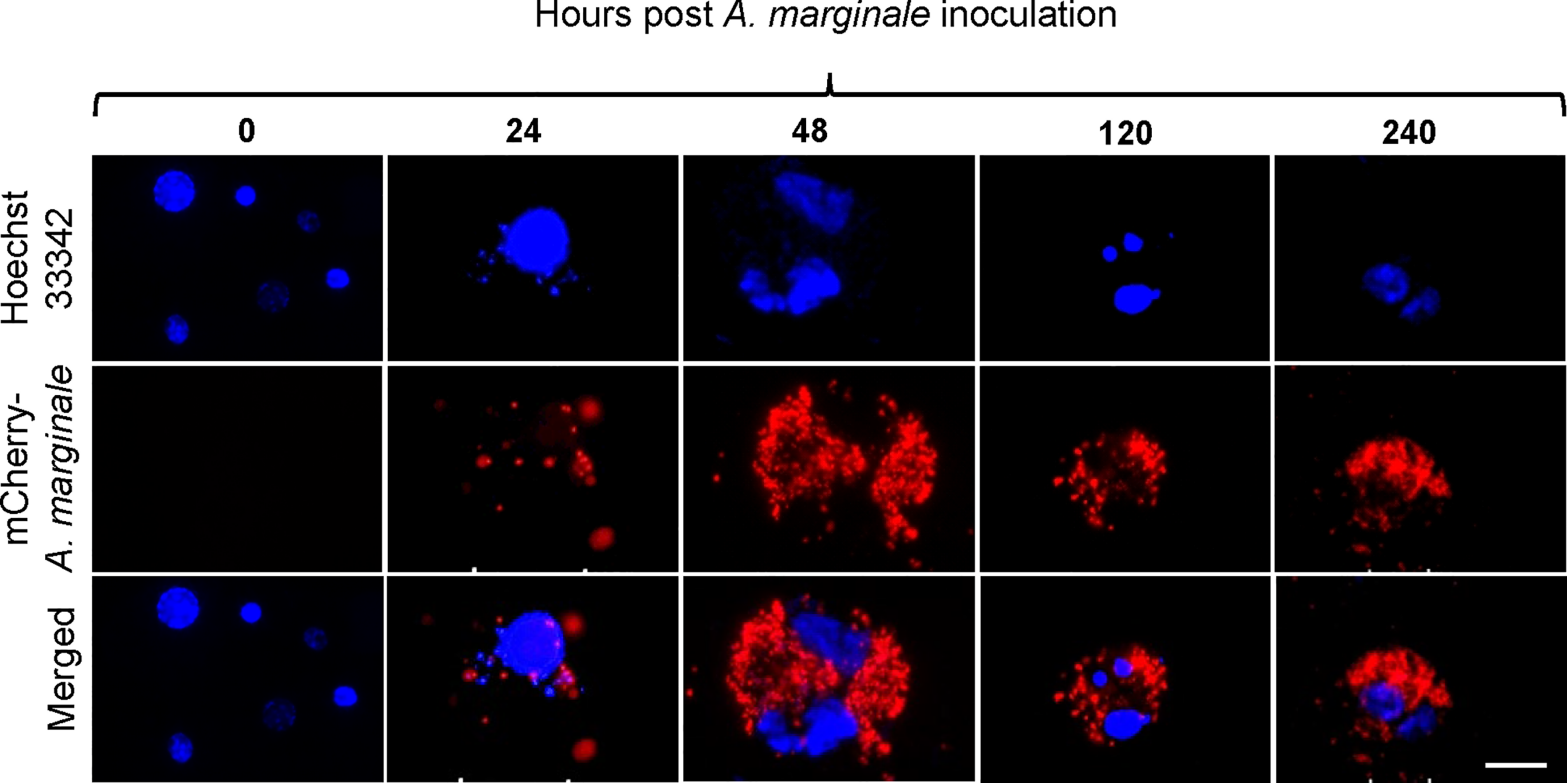
Infection of *Dermacentor andersoni* primary midgut cells by *Anaplasma marginale omp10::himar1*. *Anaplasma* colony formation was recorded from 0 h to 10 days post infection. Blue: Hoechst 33342 stained nuclei and Red: colonies of *Anaplasma marginale*. Scale bar: 10µm.

**Figure 3:**
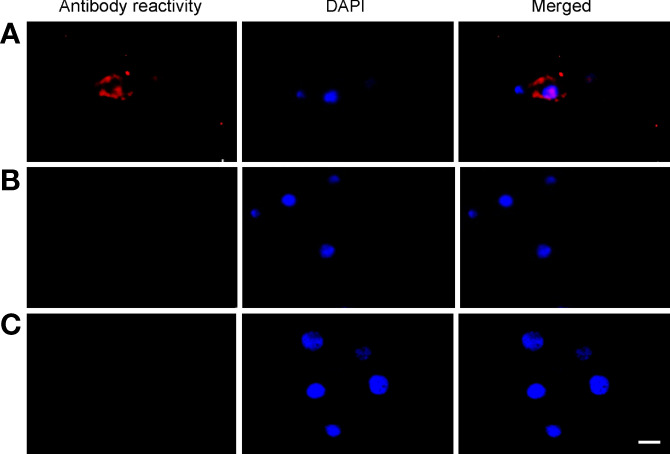
Confirmation of *A. marginale* infection using immunofluorescence of primary midgut cell cultures. Midgut cell cultures infected with *A. marginale omp10::himar1* and probed with **(A)** anti-Msp2 antibody or **(B)** Tryp 1a, an isotype control. **(C)** Uninfected midgut cell culture probed with anti-Msp2 antibody. Blue: DAPI stained nuclei and Red: Goat anti-mouse conjugated with Alexa Fluor 594. Scale bar: 10µm.

Primary midgut cell cultures were inoculated with an mCherry-expressing transformant of *A. marginale omp10::himar1* and maintained without antibiotic/antimycotic throughout all experiments. Colonies of *A. marginale omp10::himar1* were observed as early as 24 h and up to 10 days post-infection, indicating that *A. marginale* replicated in cultured primary midgut cells (**Figure 2**). Both the non-digestive like and digestive cells were susceptible to *A. marginale* infection. Colonization of *A. marginale* within primary midgut cells was confirmed using monoclonal antibody (mAb) AnaR49A1 against *A. marginale* Msp2 (**Figure 3**, panel A). No reactivity was observed in infected cells probed with isotype control, mAb Tryp 1a against a *T. brucei* protein, or in uninfected midgut cells probed with mAb AnaR49A1 (**Figures 3**, panels B and C).

### Identification of *D. andersoni* Fucosyltransferases

To determine if fucosylated glycans play an important role in *A. marginale* infection of *D. andersoni* midgut cells, fucosyltransferase genes were identified *in silico*. Using TSA database of *D. variabilis* and SRA database of *D. andersoni*, six potential N-glycan modifying fucosyltransferase genes were identified. DaFucT6 is a putative α-(1,6)-fucosyltransferase, DaFucTA1 and DaFucTA2 are putative core α-(1,3)-fucosyltransferases, and DaFucTC1, DaFucTC2, and DaFucTC3 are putative α-(1,3/4)-fucosyltransferases. The GenBank accession numbers for the *D. andersoni* fucosyltransferase sequences are listed in **Table 2**.

**Table 2:** The homology of Dermacentor andersoni fucosyltransferases shared with Dermacentor variabilis, Ixodes scapularis, Drosophila melanogaster, Aedes aegypti, and Apis mellifera.

*D. andersoni* fucosyltransferases	Accession number	Closest arthropod spp.	Accession number	Amino acid identity (%)
DaFucTA6	OL791273	*D. variabilis*	GGQS01025740.1	100
		*I. scapularis*	XP_029847506.2	67.4
		*D. melanogaster*	NP_572740.1	51.7
DaFucTA1	OL791274	*I. scapularis*	XP_029829512.2	72.0
		*A. aegypti*	XP_001661902.2	54.1
DaFucTA2	OL791275	*D. variabilis*	GGQS01027527.1	98.1
		*I. scapularis*	XP_029823740.2	64.7
		*A. mellifera*	XP_016770760.1	50.5
DaFucTC1	OL791276	*I. scapularis*	XP_040071267.1	57.1
		*A. aegypti*	XP_001660128.1	38.2
DaFucTC2	OL791277	*D. variabilis*	GGQS01025645.1	98.1
		*I. scapularis*	XP_029837352.2	63.0
		*D. melanogaster*	NP_001036320.3	37.6
DaFucTC3	OL791278	*D. variabilis*	GGTZ01014433.1	99.4
		*I. scapularis*	XP_002415567.3	56.6
		*A. mellifera*	XP_006565206.1	38.5

**Figure 4:**
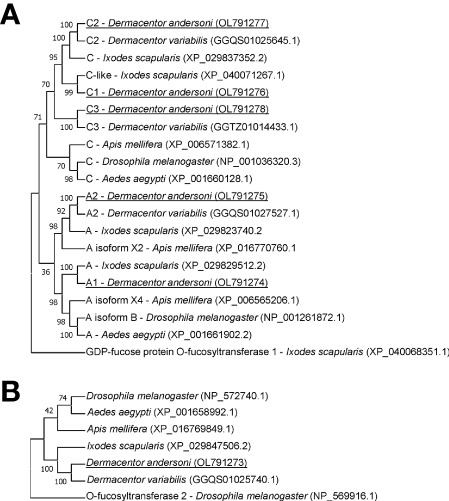
Phylogenetic analysis of fucosyltransferases from representative species of arthropods. **(A)** The phylogenetic tree of alpha-(1,3)-fucosyltransferases using the O-fucosyltransferase 1 protein of *Ixodes scapularis* as an out-group. **(B)** The phylogenetic tree of alpha-(1,6)-fucosyltransferases using the O-fucosyltransferase 2 protein of *Drosophila melanogaster* as an out-group. Bootstrap values were obtained by the neighbor-joining method (1000 replications) using the MEGA 11 software. Fucosyltransferases of *Dermacentor andersoni* reported in this study are underlined.

The amino acid sequence identity of *D. andersoni* fucosyltransferases to previously described enzymes for *Aedes aegypti*, *Apis mellifera*, *D. melanogaster*, and *Ixodes scapularis* was determined (**Table 2**). Phylogenetic comparison of fucosyltransferases of *D. andersoni* demonstrated the relationship with other arthropod fucosyltransferases (**Figure 4**).

### *Anaplasma marginale* Infection Impacted the Expression of Core α-(1,3)-Fucosyltransferases, DaFucTA1 and DaFucTA2

**Figure 5:**
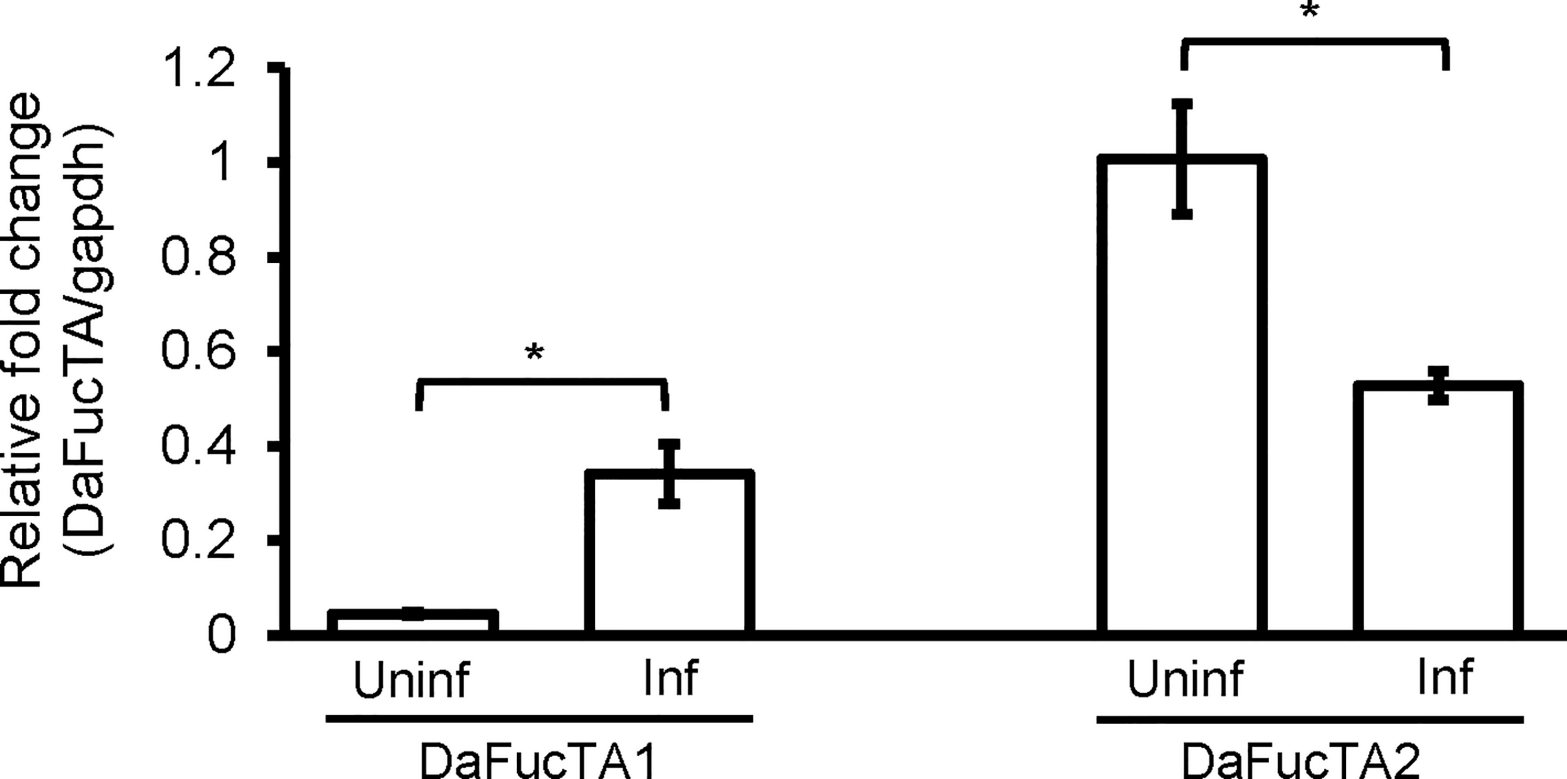
*Anaplasma marginale* infection modulates the expression of *Dermacentor andersoni* fucosyltransferases. After *A. marginale* infection, DaFucTA1 expression is up-regulated while DaFucTA2 expression is down-regulated. Primary midgut cultured cells were infected with *A. marginale omp10::himar1*. Standard error bars are shown and the lines above each gene with an asterisk denotes a *p* < 0.05 difference in gene expression as calculated with unpaired t-test.

The expression of core α-(1,3)-fucosyltransferases, DaFucTA1 and DaFucTA2, in primary midgut cells was quantified in uninfected and infected cells at 24 h post *A. marginale omp10::himar1* infection. In uninfected primary midgut cells, the relative expression of DaFucTA1 was significantly lower than DaFucTA2 (**Figure 5**). During *A. marginale omp10::himar1* infection, the relative expression of DaFucTA1 was upregulated by 7.6X fold (*p*<0.05) while DaFucTA2 was downregulated by 1.9X fold (*p*<0.01) (**Figure 5**).

### The Expression of DaFucTA1 Was Not Affected by dsRNA Mediated Gene Knockdown of DaFucTA2

**Figure 6:**
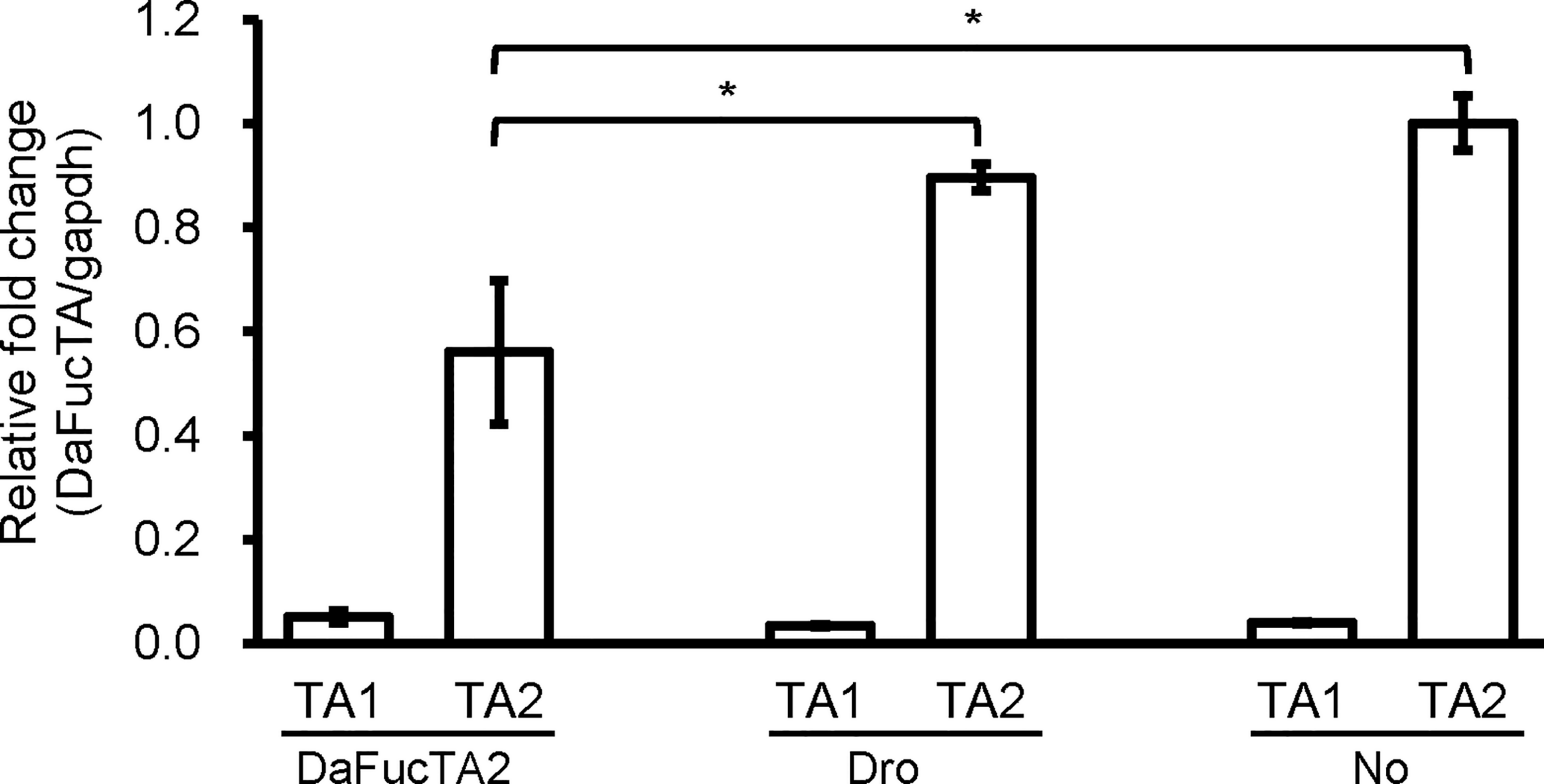
The expression of DaFucTa1 in cultured *Dermacentor andersoni* midgut cells was not affected by dsRNA mediated gene knockdown of DaFucTA2. There was a significant reduction of DaFucTA2 expression. There were no differences in DaFucTA1 expression by either dsRNA-TA2 or controls. dsRNA-Dro: non-tick specific *Drosophila nautilus* dsRNA, No dsRNA: dsRNA elution buffer + transfection reagent. Standard error bars are shown and the lines above each gene with an asterisk denotes a p < 0.05 difference in gene expression as calculated with unpaired t-test.

To determine if upregulation of DaFucTA1 expression observed during *A. marginale* infection was associated with the reduction of DaFucTA2 expression, dsRNA mediated gene knockdown of DaFucTA2 was performed. DaFucTA2 expression was significantly lower in cells silenced with dsRNA-DAFucTA2 than in the control groups (*p*<0.05) (**Figure 6**). Reduction of DaFucTA2 expression (1.8X fold) by dsRNA mediated knockdown did not affect the expression of DaFucTA1. There were no significant differences in DaFucTA1 expression between DaFucTA2 silenced and control groups (**Figure 6**).

### Silencing of DaFucTA2 Reduced the Cellular Display of Core α-(1,3)-Fucose of N-glycans and *A. marginale* Infection

**Figure 7:**
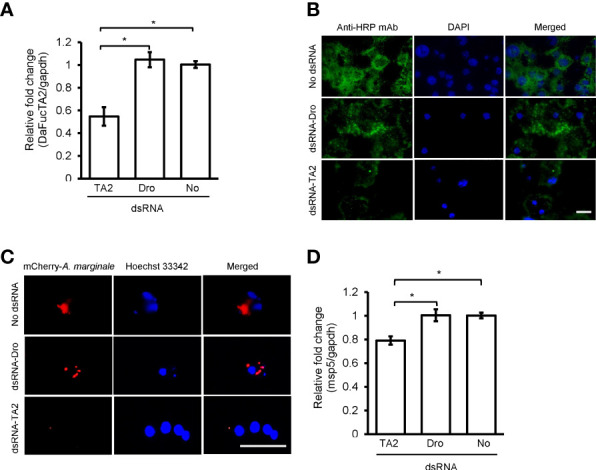
dsRNA mediated DaFucTA2 knockdown reduced the cellular display of core α (1,3)-fucose of N-glycans and *Anaplasma marginale* infection in cultured *Dermacentor andersoni* midgut cells. **(A)** DaFucTA2 gene expression after knockdown using dsRNA-TA2, dsRNA-Dro: non-tick specific *Drosophila* nautilus dsRNA, or No dsRNA: dsRNA elution buffer + transfection reagent. **(B)** Fixed immunofluorescence of primary midgut cell cultures after DaFucTA2 knockdown. Green: Detection of anti-HRP antibody binding to core α 1,3-fucose of N-glycans by goat anti-mouse IgG1 CF™488A. Blue: DAPI stained nuclei. Scale bar: 20µm. **(C)** Live imaging of infected cells. Red: Colonies of *A. marginale omp10::himar1.* Blue: Hoechst 33342 stained nuclei. Scale bar: 20µm. **(D)** Quantitative detection of *A. marginale omp10::himar1* replication following DaFucTA2 silencing. Relative fold change *A. marginale msp5* at 24h post infection. Standard error bars are shown and the lines above each gene with an asterisk denotes a p < 0.05 difference in gene expression as calculated with unpaired t-test.

Silencing the expression of DaFucTA2 by 1.8X fold (**Figure 7A**) reduced the display of core α-(1,3)-fucose by primary midgut cells as compared to control groups, dsRNA-Dro and No-dsRNA treated cells. Immunofluorescence demonstrated a reduction in the display of core α-(1,3)-fucose by primary midgut cells silenced with dsRNA-DaFucTA2 (**Figure 7B**). In contrast, the display of core α-(1,3)-fucose by control groups, dsRNA-Dro and No-dsRNA, were unchanged. The *A. marginale omp10::himar1* infection of midgut cells following DaFucTA2 silencing was investigated. Live imaging of DaFucTA2 silenced cells at 18 h post-infection showed reduced *A. marginale omp10::himar1* replication with fewer infected midgut cells containing smaller *A. marginale* colonies as compared to control cells (**Figure 7C**). The reduction of *A. marginale omp10::himar1* replication was confirmed by qPCR. The relative fold change of total *A. marginale omp10::himar1* in DaFucTA2 silenced cells at 24 h post-infection was significantly lower (1.3X fold) than controls (**Figure 7D**).

## Discussion

Tick vectors and pathogens have co-evolved molecular mechanisms for interactions (Kazimírová and Štibrániová, 2013). Successful establishment of infection by the pathogen requires adhesion to the midgut cell which is key for subsequent cellular invasion, intracellular multiplication, and dissemination to other tick organs, including salivary glands for the successful transmission of the pathogen. A cell culture system derived from tick midgut tissue is crucial to address the role of tick fucosylated glycans for the infection of midgut cells (Salata et al., 2021). Attachment to the host cell *via* glycans is a common strategy employed by bacteria and viruses during the establishment of an infection within the host (Ilver et al., 1998; Pedra et al., 2010; Chen et al., 2011; Varki, 2017; Heim et al., 2019; Suwandi et al., 2019). Multiple carbohydrate-protein interactions have been demonstrated for vector-borne pathogens (Dinglasan and Jacobs-Lorena, 2005). Using *in silico* approaches, we identified genes encoding fucosyltransferases in *D. andersoni* ticks named DaFucT6, DaFucTA1, DaFucTA2, DaFucTC1, DaFucTC2, and DaFucTC3. DaFucT6 contained an α-(1,6)-fucosyltransferase domain which is the catalytic domain containing GDP-fucose binding sites and the Src homology 3 (SH3) domain whose function is unknown. DaFucTA1, DaFucTA2, DaFucTC1, DaFucTC2, and DaFucTC3 each contained a fucosyltransferase domain and a glycosyltransferase family 10 domain. DaFucTA1 contained an additional domain, DNA polymerase III subunits gamma and tau, the functionality of which is unclear.

The isolated midgut cells were morphologically similar to the cell types previously described in the whole tick midgut (Sonenshine, 1993). The midgut cell suspension obtained following dissociation steps was used to establish primary cultures. We utilized Hink’s TNM-FH insect medium which was previously used to culture *Pseudaletia unipuncta* midgut cells (Garcia et al., 2001). We used Hink’s TNM-FH insect medium, pH 7, that mimicked the pH of unfed male *D. andersoni* hemolymph. While disrupting intercellular connections, it was difficult to balance cell viability with cell dissociation. In previous studies, collagenase was used to disrupt tissue integrity (Garcia et al., 2001; Mosqueda et al., 2008). Collagenase type XI functions by cleaving triple-helical bonds in collagen (Chung et al., 2004). The DTT was used to gain single cells by disrupting disulfide bonds (Gracz et al., 2012). We used fetal bovine serum to stabilize the cell viability during the enzyme digestion (Feng et al., 2018). Previous studies used 20-HE to promote embryonic or differentiated insect cell growth and differentiation (Sadrud-Din et al., 1994, Hakim et al., 2009). Also, it has been documented that 2 and 20 µM significantly suppressed growth of RAE25 and ANE58 cell lines derived from *Rhipicephalus appendiculatus* and *Dermacentor nitens*, respectively (Kurtti and Munderloh, 1983). The suppression of growth for the young tick cell line ANE58 was determined to be 20-HE dose-dependent. In the ANE58 cell line, the suppression of growth by 20-HE was less evident than for RAE25. Further investigation is required to test the optimum concentration of 20-HE. Additionally, growth promoters such as heparin (Flint et al., 1994), epidermal growth factor, platelet-derived growth factor (Booth et al., 1995; Loeb et al., 2003), retinoic acid (Loeb et al., 2003), and transferrin (Ali and Reynolds, 1996) should be tested for their ability to stimulate cell proliferation in primary cultures of *D. andersoni* midgut cells.

We studied two fucosyltransferases, DaFucTA1 and DaFucTA2 that are accountable for the addition of core α-(1,3)-fucose to the N-glycans. Phylogenetic analysis showed that both genes have high amino acid sequence identity with other arthropod species. Homologs of these two fucosyltransferases found in *Ixodes* spp. were previously studied and recognized as enhancing *A. phagocytophilum* infection in whole *I. scapularis* ticks and an *I. ricinus* IRE/CTVM19 cell line (Pedra et al., 2010). We demonstrated the display of core α-(1,3)-fucose by *D. andersoni* midgut primary cell culture by using antibody staining. We used polyclonal antibodies raised against a plant glycoprotein, horseradish peroxidase (HRP), that cross-react with core α-(1,3)-fucosylated N-glycans of arthropod tissues (Wilson et al., 1998; Fabini et al., 2001). Both DaFucTA1 and DaFucTA2, are responsible for the addition of core α-(1,3)-fucose to N-glycans. The expression level of DaFucTA1 by uninfected cells was negligible compared to DaFucTA2 suggesting the latter normally plays the prominent role in the addition of core α-(1,3)-fucose. However, the expression of both genes was inversely affected during *A. marginale* infection indicating changes to tick cell gene regulation in response to the bacterium. The upregulation of DaFucTA1 expression may be compensation for the downregulation of DaFucTA2. Nevertheless, silencing of DaFucTA2 did not increase the expression of DaFucTA1, suggesting that DaFucTA1 expression is independent of DaFucTA2 or that the timing or level of silencing was not sufficient to trigger a response.

Genes in tick cell lines are easily silenced using dsRNA, often not requiring any assistance from transfection reagents or electroporation (Blouin et al., 2008; Barry et al., 2013). In this study, we used dsRNA to silence gene expression in primary tick midgut cell cultures and achieved a knockdown efficiency of ~45%. This knockdown efficiency is comparable to a previous study using a tick cell line that showed knockdown efficiencies between 31-100% for 10 different genes (Kurscheid et al., 2009). The reduction of intracellular *A. marginale* replication at 18 h post-infection during the knockdown of DaFucTA2 was well documented using live microscopic images. The reduction of *A. marginale* levels measured by qPCR at 24 h post-infection during DaFucTA2 knockdown was significantly greater than in the control. We recognize that *A. marginale* utilizes core α-(1,3)-fucose of midgut cells for infection. There is a need to identify midgut surface proteins containing core α-(1,3)-fucosylated N-glycans. These surface proteins should be tested as potential vaccine candidates to prevent *A. marginale* transmission by the tick vectors. There is a possibility that gene knockdown affected cell viability which may have resulted in a reduction of pathogen infection. In this study cell viability was not directly measured after DaFucTA2 knockdown. Instead, we used *gapdh* transcript levels as a surrogate readout for cell viability. Previous studies have used mRNA to estimate cell viability within a population *in vitro* (Wong et al., 2020; Collins et al., 2022). We showed that there were no significant differences (*p*>0.05) in the *gapdh* cycle threshold value between mRNA obtained from an equal number of DaFucTA2 silenced or control cells (**Supplementary Figure 2**) indicating a similar number of viable cells was present in each treatment group.

In conclusion, we developed a primary cell culture system derived from *D. andersoni* tick midgut cells and investigated the molecular interaction between tick midgut cells and *A. marginale*. Primary tick midgut cells were permissive for *A. marginale* infection and required surface proteins containing core α-(1,3)-fucosylated N-glycans for infection. We also identified *D. andersoni* fucosyltransferases and demonstrated that, upon *A. marginale* infection, DaFucTA1 expression was upregulated while DaFucTA2 was downregulated. This is the first step in understanding the importance of sugar moieties for *A. marginale* interaction with midgut cells of biological tick vectors, which may address future application for the development of new interventions for pathogen transmission.

## Data Availability Statement

The original contributions presented in the study are included in the article/**Supplementary Material**, further inquiries can be directed to the corresponding author.

## Author Contributions

RV, JC-P, WJ, HH, KB, SN, and MU conceived the experiments, RV, JC-P, and MU conducted the experiments, RV, WJ, NT, KB, UM, SN, and MU analyzed the results, RV and MU wrote the original draft, WJ, NT, JC-P, HH, KB, UM, and SN, revised and edited. All authors contributed to the article and approved the submitted version.

## Funding

This work was supported by Agricultural Research Service Project #2090-32000-041-00D.

## Conflict of Interest

The authors declare that the research was conducted in the absence of any commercial or financial relationships that could be construed as a potential conflict of interest.

## Publisher’s Note

All claims expressed in this article are solely those of the authors and do not necessarily represent those of their affiliated organizations, or those of the publisher, the editors and the reviewers. Any product that may be evaluated in this article, or claim that may be made by its manufacturer, is not guaranteed or endorsed by the publisher.
