# Supplementary material for: Anaplasma marginale Infection of Dermacentor andersoni Primary Midgut Cell Culture Is Dependent on Fucosylated Glycans
Source: Front Cell Infect Microbiol. 2022 May 31;12:877525. doi: 10.3389/fcimb.2022.877525 (PMC9197492; doi:10.3389/fcimb.2022.877525)
Supplement: Supplementary file 1 [file DataSheet_1.pdf]

TSA and SRA alignments

↓

Determine Putative open reading frames

↓

Design primers

↓

Amplify *D. andersoni* midgut cDNA

↓

Clone and extract plasmid DNA

↓

Sequence and analyze

Supplemental Figure 1. A flow diagram showing the process used to identify *Dermacentor andersoni* putative genes.

(A) (B)

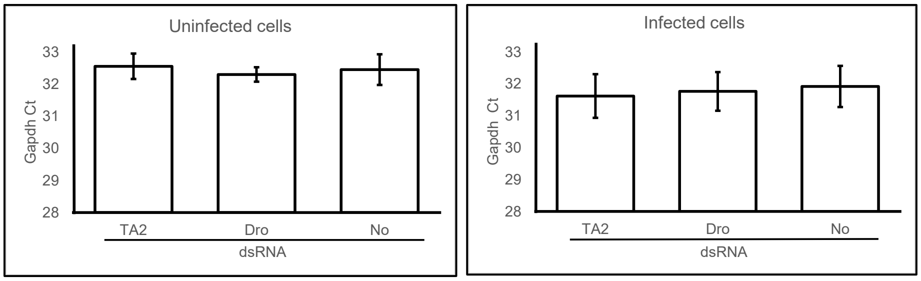

Supplemental Figure 2. Measurement of *gapdh* transcripts. The *gapdh* cycle threshold value of cells from DaFucTA2 silenced and control groups (A) without *A. marginale* infection and (B) with *A. marginale* infection. Standard errors bars are shown. There were no significant differences (p>0.05) in *gapdh* ct values between an equal number of cells from DaFucTA2 silenced or control groups.
